# Harnessing Fermented Soymilk Production by a Newly Isolated *Pediococcus acidilactici* F3 to Enhance Antioxidant Level with High Antimicrobial Activity against Food-Borne Pathogens during Co-Culture

**DOI:** 10.3390/foods13132150

**Published:** 2024-07-07

**Authors:** Sitha Chan, Kaemwich Jantama, Chutinun Prasitpuriprecha, Supasson Wansutha, Chutchawan Phosriran, Laddawan Yuenyaow, Kuan-Chen Cheng, Sirima Suvarnakuta Jantama

**Affiliations:** 1 Faculty of Pharmaceutical Sciences, Ubon Ratchathani University, Warinchamrap, Ubon Ratchathani 34190, Thailand; sithachan34@gmail.com (S.C.); chutinun.p@ubu.ac.th (C.P.); supasson.wa@udru.ac.th (S.W.); laddawan.yu.59@ubu.ac.th (L.Y.); 2 School of Biotechnology, Institute of Agricultural Technology, Suranaree University of Technology, Muang, Nakhon Ratchasima 30000, Thailand; chutchawan.p@nsru.ac.th; 3 Faculty of Science, Udon Thani Rajabhat University, Udon Thani 41000, Thailand; 4 Institute of Biotechnology, College of Bioresources and Agriculture, National Taiwan University, Taipei 10617, Taiwan; kccheng@ntu.edu.tw; 5 Institute of Food Science and Technology, College of Bioresources and Agriculture, National Taiwan University, Taipei 10617, Taiwan; 6 Department of Optometry, Asia University, 500, Lioufeng Rd., Wufeng, Taichung 41354, Taiwan; 7 Department of Medical Research, China Medical University Hospital, China Medical University, 91, Hsueh-Shih Road, Taichung 404327, Taiwan

**Keywords:** antioxidation, *Pediococcus acidilactici*, soymilk, antimicrobial activity, probiotics

## Abstract

In this study, a newly isolated *Pediococcus acidilactici* F3 was used as probiotic starter for producing fermented soymilk to enhance antioxidant properties with high antimicrobial activity against food-borne pathogens. The objectives of this study were to investigate optimized fermentation parameters of soymilk for enhancing antioxidant property by *P. acidilactici* F3 and to assess the dynamic antimicrobial activity of the fermented soymilk during co-culturing against candidate food-borne pathogens. Based on central composite design (CCD) methodology, the maximum predicted percentage of antioxidant activity was 78.9% DPPH inhibition. After model validation by a 2D contour plot, more suitable optimum parameters were adjusted to be 2% (*v*/*v*) inoculum and 2.5 g/L glucose incubated at 30 °C for 18 h. These parameters could provide the comparable maximum percentage of antioxidant activity at 74.5 ± 1.2% DPPH inhibition, which was up to a 23% increase compared to that of non-fermented soymilk. During 20 days of storage at 4 °C, antioxidant activities and viable cells of the fermented soymilk were stable while phenolic and organic contents were slightly increased. Interestingly, the fermented soymilk completely inhibited food-borne pathogens, *Salmonella* Typhimurium ATCC 13311, and *Escherichia coli* ATCC 25922 during the co-culture incubation. Results showed that the soymilk fermented by *P. acidilactici* F3 may be one of the alternative functional foods enriched in probiotics, and the antioxidation and antimicrobial activities may retain nutritional values and provide health benefits to consumers with high confidence.

## 1. Introduction

Interest in soy-based foods increased after the growing demand for alternatives to dairy products due to problems with lactose intolerance and allergy to cow’s milk, as well as the increased demand for vegan products. In the year 2021, about 42% of global consumers preferred to choose plant-based products and about USD 18 billion in 2021 came from plant-based milk [1]. Soy is considered as one of the most beneficial functional foods because it is rich in high-quality proteins. It contains no lactose and no cholesterol and a small quantity of saturated fatty acids. Soy-based products are also suitable for people who suffer from lactase deficiency and hyperlipidemia. Additionally, soy products provide phenolic compounds exhibiting high antioxidant activity. Fermentation of soybean using lactic acid bacteria (LAB) yielded higher crude and insoluble protein contents, released free isoflavone contents, and provided small peptides containing antioxidant activities [2]. In soy-fermented products, the antioxidant substances have been claimed to mainly consist of antioxidant enzymes, manganese ions, mercapto-compounds, bioactive peptides, and exopolysaccharides (EPSs), providing great health benefits by preventing or postponing the onset of degenerative diseases in which natural antioxidants function in several ways including preventing the formation of radicals, scavenging free radicals, hydrogen peroxide, and other peroxides. Even though many published literature studies revealed that the production of soymilk fermented by the extensive use of only *Lactobacillus* and *Bifidobacterium* species [2] enhanced its antioxidant activity, the information about nutritional properties of soy-fermented products are limited within both species. The development of soymilk fermented by other newly isolated LAB species as an alternative probiotic stater should be more investigated to gain broader knowledge for further applications in soy-fermented products or other fermented plant-based products produced by alternative beneficial LAB species. *Pediococcus acidilactici* is known as one of the beneficial and safe species in the context of food and livestock microbiology [3] and can act as a probiotic starter [4]. Even though the application of *Pediococcus* species in fermented dairy products is restricted by their inability to rapidly ferment lactose, *P. acidilactici* has several advantages when administrating it as a food biopreservative since it produces an antimicrobial peptide (pediocin) with anti-listerial activity. *P. acidilactici* is also resistant to heat, cold, pH, and proteolytic treatments, thus making it suitable for a broad range of food applications [5]. To date, only two publications reported the use of *P. acidilactici* to produce fermented soymilk, though only physicochemical changes and nutritional values in soymilk matrices and sensory evaluation were reported [6,7]. The information regarding antimicrobial activity against food-borne pathogens of the finished product is still missing. Additionally, the suitable condition for soymilk fermentation has not yet been investigated and reported.

Recently, the *P. acidilactici* F3 strain was newly isolated from the local fermented fish in Thailand. The F3 isolate showed great performance in antimicrobial activity against food-borne pathogens and *Candida* sp. [8]. It is likely that this strain may exhibit excellent probiotic characteristics for the protection of humans against pathogen infections since it tolerates high acidic conditions and high bile salt concentrations when it is administered to the GI tract. Based on the preliminary results of Chan et al. [9], the F3 strain offered higher antioxidant properties when it was used as a starter for fermenting soymilk compared to those of some industrially used LAB species under uncontrolled conditions. However, optimized parameters providing the highest antioxidant level during soymilk fermentation by the F3 strain have not yet been studied. If applicable, this information is mandatorily required to produce the fermented soymilk on a commercial scale. To elucidate a further possibility to utilize this microbe in plant-based products, the objectives of this study were to investigate the optimized fermentation parameters of soymilk for enhancing antioxidant property by *P. acidilactici* F3 and to assess the dynamic antimicrobial activity of the fermented soymilk during co-culturing against candidate food-borne pathogens, including *Escherichia coli* ATCC 25922, *Salmonella* Typhimurium ATCC 13311, and *Staphylococcus aureus* ATCC 25923. In addition, antioxidant and antimicrobial activities of the fermented soymilk for an entire shelf-life storage were elucidated to ensure the product’s stability. The results revealed that the levels of antioxidant and antimicrobial properties were significantly enhanced in the fermented soymilk under optimized conditions. These properties were also stable during storage at 4 °C for 20 days. Our results therefore demonstrated the potential insight of fermented soymilk as a vehicle delivering the *P. acidilactici* F3 strain to promote health benefits in consumers concerning the ingestion of plant-based products as one of the alternative functional foods.

## 2. Materials and Methods

### 2.1. Bacterial Strains

A new *P. acidilactici* F3 probiotic strain [8] isolated from fermented fish was kept and maintained at −80 °C in the culture collection facility at the Faculty of Pharmaceutical Sciences, Ubon Ratchathani University, Thailand, with the accession number UBU-LAB-PA-F3. All microorganisms used in this study were initially streaked on de Man Rogosa and Sharpe (MRS) agar and incubated for 37 °C for 24 h. A single colony for each strain was inoculated in 4 mL in MRS broth and incubated at 37 °C for 18 h without stirring under micro-aerobic conditions. Inoculum was adjusted to an optical density (OD) at 600 nm of 0.75 by using a spectrophotometer before inoculation into MRS medium or soymilk fermentation. The correlation of reading OD_600_ and CFUs/mL of LAB strains in MRS broth was obtained by plotting viable cell counts against cultures’ densities. Approximately, an initial OD_600_ at 3.0 in MRS broth was equivalent to 2.7 × 10^9^ CFUs/mL.

### 2.2. Culture Conditions

A pasteurized and non-sugar-added soymilk was purchased from the local supermarket (OHAYO™, Pathumthani, Thailand). The soymilk contained 2% (*w*/*v*) fat, 4% (*w*/*v*) protein, 1.5% (*w*/*v*) carbohydrate, and 2% (*w*/*v*) fiber. For soymilk fermentation, one hundred milliliters of soymilk were inoculated with 1% (*v*/*v*) active inoculum at an OD_600_ of 0.75 and cultures were allowed to ferment at 37 °C for 24 h. Samples were aseptically withdrawn at 24 h and immediately stored in a refrigerator. Additionally, non-inoculated soymilk incubated under the same experimental conditions was used as a control. Experiments were performed in triplicate.

### 2.3. Optimization of Fermentation Parameters by Response Surface Methodology (RSM)

RSM methodology was employed to determine the optimized parameters of inoculum size, temperature, fermentation time, and glucose concentration with different levels (Table 1) for enhancing the antioxidant activity of soymilk fermented by the *P. acidilactici* F3 strain. Design expert program (version 8.0) was used to iterate the central composite design (CCD) to maximize the response of the percentage of antioxidant activity. All fermentation experiments were performed in 200 mL shake flasks with a total volume of 100 mL in triplicate.

### 2.4. Preparation of Fermented Soymilk Extract

Fermented soymilk samples (10 g) were homogenized with 2.5 mL of sterile distilled water and the homogenates were acidified to pH 4.0 with 1 M HCl followed by heating at 45 °C for 10 min. The samples were centrifuged at 4000 rpm for 10 min at 4 °C. The pH of supernatants was adjusted to 7.0 using 3 M NaOH, and the supernatants were re-centrifuged for the further precipitation of proteins and salts. The supernatants were harvested and kept in the refrigerator (4 °C) and used for further analysis within 12 h.

### 2.5. Storage Stability

After 18 h of fermentation, the fermented soymilk was stored in sterile flasks at 4 °C for 0, 5, 10, 15, and 20 days. These fermented soymilk products were assessed for total phenolic content, a percentage of 2,2-diphenyl-1-picrylhydrazyl (DPPH) radical scavenging activity, viable cell counts, pH, and organic acid contents. Soymilk without fermentation was used as a control. All experiments were performed in triplicate.

### 2.6. Antimicrobial Activity by Co-Culture Incubation

*Escherichia coli* ATCC 25922, *Salmonella* Typhimurium ATCC 13311, and *Staphylococcus aureus* ATCC 25923 were obtained from the culture collection facility at the Faculty of Pharmaceutical Sciences, Ubon Ratchathani University, Thailand. These pathogens were separately added to fermented soymilk samples incubated at different storage times. The inoculum of each pathogen was prepared by inoculating seed culture into 4 mL Tryptic soy broth (TSB) at 37 °C for 16 h. Cell inoculum was diluted to an OD_600_ of 0.1 prior to inoculating 1 mL of inoculum into 100 mL of each fermented soymilk sample (D0, D5, D10, D15, and D20) or non-fermented soymilk. The cultures were further incubated at 37 °C for 24 h. Serial ten-fold dilutions of the incubated cultures were conducted before plating. The diluted samples of 100 µL were spread on selective agar plates. *E. coli* ATCC 25922, *S.* Typhimurium ATCC 13311, and *S. aureus* ATCC 25923 counts were estimated by inoculating cultures at the suitable dilution on MacConkey Agar, Salmonella Shigella agar, and mannitol salt agar, respectively. The viable count of each strain was expressed as CFUs/mL by multiplying the number of cells grown on the plate with the corresponding dilution factor and this was divided by the volume of the culture plated.

### 2.7. Analytical Methods

#### 2.7.1. 2,2-Diphenyl-2-picryl-hydrazyl (DPPH) Assay

One milliliter of MRS broth or extracted samples and 0.8 mL of 0.2 mM DPPH ethanol solution were mixed and kept in dark conditions for 30 min. The absorbance of reaction mixtures was measured at 517 nm. Ethanol and MRS medium were used as the blank and control treatments, respectively. The percentage of DPPH free radical scavenging activity was calculated using a method adapted from Heo et al. [10] with the following equation.

DPPH radical scavenging activity (%) = [1 − (A_517_ sample/A_517_ control)] × 100 (1)

#### 2.7.2. Phenolic Content

Total phenolic content was measured by Folin–Ciocalteau reagent. The method was adapted from Livinska et al. [11]. An aliquot (1 mL) of extract samples was introduced into a test tube with 5 mL of Na_2_CO_3_ at 7% (*w*/*v*) solution and was mixed briefly with 1 mL Folin–Ciocalteu reagent. The solution was stirred and allowed to stand for 30 min. The absorbance of the supernatant was measured at 725 nm and data were expressed as micrograms gallic acid equivalent (GAE)/g (Sigma-Aldrich, Buchs, Switzerland). GAE at 0.5 g was dissolved into 10 mL ethanol and adjusted to 100 mL in a volumetric flask using water. Serial dilutions of the GAE standard were 50, 100, and 150 mg/L.

#### 2.7.3. Organic Acid Measurement

Organic acids and sugars were determined by using high-performance liquid chromatography, HPLC (Agilent Technology 1200 series, Frankfurt, Germany), equipped with refractive index detectors with a Bio-Rad Aminex HPX-87H ion exclusion column. The column and detector temperatures were kept constant at 45 °C. The mobile phase used in the HPLC system was 4 mM sulfuric acid at a flow rate of 0.4 mL/min. One gram of fermented samples was mixed with 1 mL sterile water. The mixtures were thoroughly mixed and were centrifuged at 13,500 rpm for 4 min. The supernatant was filtered through a 0.2 μm filter membrane before injection to HPLC. An injection volume of ten microliters was used for analysis.

### 2.8. Statistical Analysis

The mean values and the standard deviations were expressed from data obtained from triplicate experiments. Differences between sample means were analyzed by Duncan’s Multiple Range tests with a level of significance at 95% by SPSS software version 17.0.

## 3. Results and Discussion

### 3.1. Optimization of Fermented Soymilk Enhancing Antioxidant Level by P. acidilactici F3

Parameters including glucose concentration, inoculum size, fermentation time, and temperature were optimized for the highest level of antioxidant activity produced by *P. acidilactici* F3 in fermented soymilk using central composite design (CCD) (Table 2). Based on the result of CCD, the fitted equation (R) of the percentage of antioxidant activity was constructed. Parameters including inoculum size (A) at 2% (*v*/*v*), temperature (B) at 30 °C, fermentation time (C) of 27.3 h, and glucose concentration (D) of 5 g/L were proposed as the optimal conditions for fermenting soymilk with *P. acidilactici* F3, thus yielding the highest observed level of antioxidant activity at 81.1 ± 0.2%. The maximum percentage of antioxidant activity was calculated at the level of 78.9% DPPH inhibition by the following fitted equation (R):

R = 80.08 − 0.72 × A + 1.06B + 0.74 × C − 0.96 × D − 1.93 × AB − 0.75 × AC − 2.93 × AD + 0.14 × BC − 2.72 × BD + 1.21 × CD − 3.08 × A^2^ − 3.50 × B^2^ − 1.11 × C^2^ − 1.88 × D^2^ (2)

From a model analysis, the model F-value of 16.00 indicates the significance of the model. Values of Prob > F less than 0.05 indicate that the model terms are significant. In this case, AB, AD, BD, CD, A^2^, B^2^, C^2^, and D^2^ are significant in the model terms. This implied that all four parameters had interaction effects on each other for an enhancement of antioxidant activity. As a result, a full quadratic second-order polynomial equation appropriately fitted the data. The lack of fit F-value of 0.31 implies that the lack of fit is not significant relative to the pure error and shows the adequacy of the model. The multiple correlation coefficients (R^2^) and adjusted R^2^ of this model are 0.91 and 0.85, respectively. This means that the model can explain more than 91% of the variability in the response and that only less than 9% of the variability is due to noise. The similarity between the R^2^ and adjusted R^2^ shows the adequacy of the model to predict the response in the optimization process.

### 3.2. Validation of the Mathematical Model

**Figure 1:**
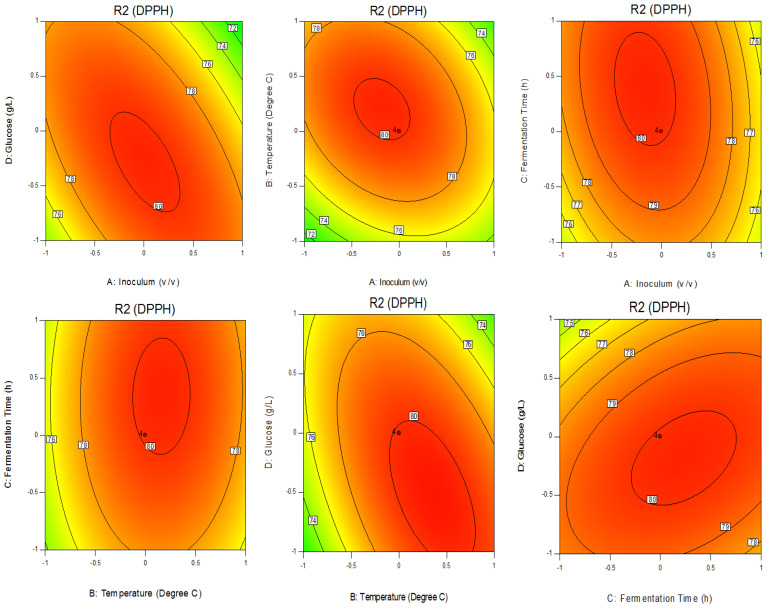
The 2D plots of the optimized parameters for %DPPH inhibition produced by *P. acidilactici* F3 in fermented soymilk.

In most previously published works, optimized parameters affecting the growth of probiotics in soymilk were assessed and investigated [12,13] without concern for the level of antioxidant activity in the finished products. In contrast, our current study directly focused on the parameters affecting the antioxidant activity during soymilk fermentation as a response. Based on CCD, the fermentation time of 27.3 h seemed not to be practical when applied to large-scale production due to a higher energy consumption. To reduce the fermentation time to be suitable for industrial applications, the optimized parameters thus also allowing the highest antioxidation activity in soymilk fermented by *P. acidilactici* F3 equivalent to those of the fitted model were re-adjusted. Based on the 2D contour plot in Figure 1, the fermentation time at 18 h with 2% (*v*/*v*) inoculum and 2.5 g/L glucose incubated at 30 °C provided an alternative option. These parameters were confirmed by our repeatable experimental runs, thus yielding an antioxidant activity around 74.5 ± 1.2% DPPH scavenging activity. Additionally, an increased level of glucose to 7.5 g/L did not only further enhance the scavenging activity up to 77.1 ± 0.9% but also shortened the fermentation time to 16 h (Table 2). There were some advantages using the short fermentation time of 16–18 h in which the energy consumption was more conserved. With those optimal conditions, the antioxidant activity of fermented soymilk was increased by 7.9–11.7% compared to that of the non-optimized conditions that required 24 h to yield an antioxidant activity of only 69.02%.

According to the optimized parameters found, the optimum temperature of 30 °C offered higher levels of antioxidant activity, while the production of antioxidant activity by the F3 strain at a higher temperature of 40 °C was slightly lower. This result indicates that incubation temperatures for producing soymilk fermented by the F3 strain can be varied from 30 °C to 40 °C with a minor effect on the antioxidant production. However, the optimum temperature of 30 °C provided more advantages over the incubation at 40 °C in which the energy consumption is lower. Hlahla et al. [14] showed that the polyphenol content significantly improved when the fermentation of bush tea commenced at 30 °C. On the other hand, they also revealed that the antioxidant activity of the fermented bush tea decreased when fermentation temperatures increased in the range of 30–42 °C. An increase in temperature up to 42 °C caused the greatest reduction in polyphenol contents in the fermented bush tea. It was likely that the antioxidant activity decreased with increasing temperature. This also suggests that fermentation at lower temperatures should be allowed to preserve the total phenolic content and antioxidant activity in finished products. However, the stability of antioxidant activity depends on the types of phenolic compounds produced by LAB strains that are still preserved in the finished products during fermentations. Reblova [15] revealed that easily oxidizable phenolic compounds such as gallic acid, gentisic acid, protocatechuic acid, and caffeic acid exhibited a slower decreasing rate of their antioxidant activity than that of less oxidizable ones including syringic, ferulic, sinapic, and vanillic acids. On the other hand, the fermentation temperature at 20 °C caused a significant reduction in the production of antioxidant activity by the F3 strain. This suggested that lower temperatures could affect the growth and metabolic activity of the F3 strain, thus resulting in a low level of antioxidant activity.

Most of previous studies revealed that soymilk alone is not a good substrate for preparing a fermented beverage with LAB strains due to low contents of sucrose and glucose, thus affecting optimal microbial growth. Therefore, glucose was supplied in this study to support growth and the ability to produce antioxidant substances of the F3 strain during soymilk fermentation. In our study, no growth and significant levels of antioxidant activity were observed when glucose was provided at concentrations of 1.5–7.5 g/L (Table 2). This suggested that a minimized glucose concentration seemed to be an acceptable level for soymilk fermentation. Glucose at 2.5 g/L was found to be the optimal concentration for soymilk fermentation to provide a highly comparable %DPPH activity even though the higher glucose concentration (7.5 g/L) resulted in only a small increase in %DPPH activity (77.1 ± 0.09%) (Table 2). Khoshayand et al. [12] showed that glucose at the higher concentration of 5.23 g/L provided the optimal growth of *L. casei* PTCC 1608 in soymilk. However, glucose concentrations at 1.5–7.5 g/L used in our study did not significantly affect the microbial growth and the production of antioxidant activity of *P. acidilactici* F3. Fortunately, the use of lower glucose concentrations in our fermented soymilk products may benefit consumers who are involved with the consumption of low-calorie products.

Additionally, the optimum fermentation time was found to be 18 h in our study. This agreed with the findings of Rui et al. [13] that the optimized fermentation duration of 18 h yielded the highest concentration of LAB cells during the solid-state fermentation of soy seed and reduced the highest level of antinutritional components including phenolics, saponin, phytic acid, and trypsin inhibitors in soybean grains. In contrast, some studies revealed that the incubation time had to be prolonged to attain desirable viable cells in fermented soymilk. Khoshayand et al. [12] used *L. casei* PTCC 1608 to ferment soymilk to obtain the highest amount of viable cells (8.23 log CFUs/mL) and antioxidant activity after prolonging the fermentation for up to 48 h. Wang et al. [16] demonstrated that *L. acidophilus* CCRC 14079 and/or *S. thermophilus* CCRC 14085 co-cultured with *Bifidobacterium infantis* CCRC 14633 and/or *B. longum* B6 were tested for their production of antioxidant scavenging activities against ascorbate autooxidation, superoxide anion radicals, and hydrogen peroxide in fermented soymilk. The incubation time of soymilk was also prolonged up to 24 h to achieve anti-scavenging effects in fermented soymilk. Zhao and Shah [17] reported that DPPH-radical scavenging efficiency reached only 30.0–36.5% by using each single culture of *L. rhamnosus* and *L. acidophilus* to ferment soymilk after 24 h. Compared to our results, it is likely that *P. acidilactici* F3 may potentially be used as a single culture for producing both a high antioxidant capacity (up to 77% scavenging activity) and viable count in fermented soymilk (up to 9.8 log CFUs/mL) within the shorter incubation time. The high viable cell counts of the F3 strain found in our fermented soymilk have met the minimal standard for commercialized probiotic products in which the viable cell count in the products has to be at least 6.0 log CFUs/mL recommended by the US FDA. To the best of our knowledge, most of the probiotics belonging to the genera of *Lactobacillus*, *Lactococcus*, *Bifidobacterium*, and *Streptococcus* were reported to be employed in food and milk beverages, in which their viable cell counts were monitored. Alternatively, our current research paid more attention and primarily studied the production of antioxidant activity by the new promising probiotic *P. acidilactici* F3 in fermented soymilk. Our work may promote further research on *P. acidilactici* applied in food industries, extending its function as one of the functional products.

### 3.3. Product Storage and Stability

Several factors provoked the instability of milk and soymilk fermented by LAB strains including viable cells, acidity, post-acidification, sensitivity to antimicrobial substances produced by starter bacteria, as well as the loss of antioxidant activity. Therefore, our research investigated some key factors affecting product stabilities during storage from the aspect of future product development.

#### 3.3.1. DPPH Scavenging Activity and Total Phenolic Content (TPC)

**Figure 2:**
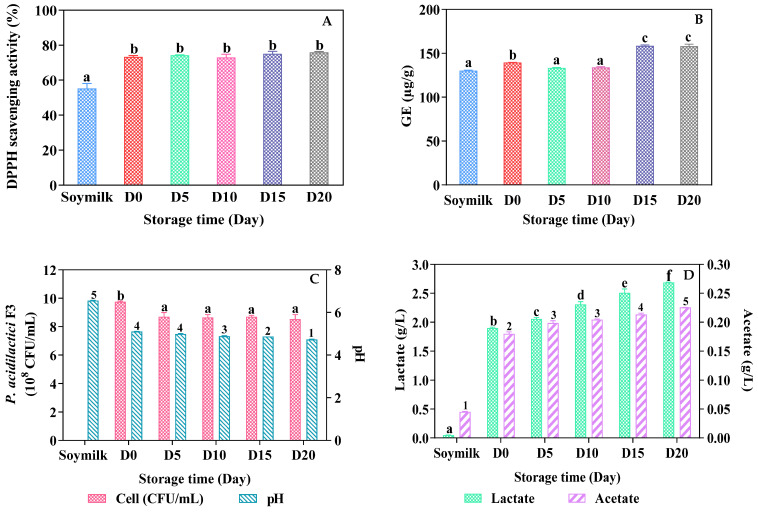
The percentage of DPPH scavenging activity (**A**), phenolic content (**B**), *P. acidilactici* F3 viable counts and pH (**C**), and organic acids production (**D**) of fermented soymilk during 20 days of storage at 4 °C. Each bar bearing different lowercase letters and numbers represents the significant difference (*p* < 0.05).

The stability of antioxidant scavenging activity was observed under the storage condition at 4 °C for 20 days as shown in Figure 2A. The percentage of DPPH scavenging activity in fermented soymilk increased by 23% after an 18 h fermentation time compared to non-fermented soymilk by *P. acidilactici* F3. Previous research demonstrated that the antioxidant activity of fermented soy foods was remarkably stronger than unfermented steamed soybeans [18]. After 20 days of storage, our results also showed that the %DPPH scavenging activity of fermented soymilk was not significantly different during the storage time. It confirmed that our fermented soymilk was stable in terms of antioxidant activity during storage for 20 days. In contrast to our study, Shori and Baba [19] found that antioxidant activities in fermented cow and camel milks in the absence or presence of the herb *Cinnamomum verum* were elevated after storage for 21 days. Hur et al. [20] suggested that fermentation has a positive influence on the total phenolic content and antioxidant activity of cereals. The degree of influence depends on the species of microorganisms.

Both antioxidant and phenolic contents were found to be higher in fermented soymilk than those of dairy yogurt due to the existence of the natural components in soymilk, thus making fermented soymilk as rich in antioxidant and phenolic contents as healthy food products. In our study, total phenolic compounds (TPCs) in soymilk increased after fermentation for 18 h by *P. acidilactici* F3 in the fermented soymilk up to 139.55 μg/g compared to non-fermented soymilk (130.73 μg/g). Further, TPCs gradually increased in fermented soymilk after storage for 15 and 20 days up to 165 μg/g, as shown in Figure 2B. Noticeably, the phenolic content of fermented soymilk increased after storage for 15 and 20 days but not before that time. It can be hypothesized that the formation/degradation of polymeric phenols may occur during phenolic and/or starch mobilization during fermentation by microorganisms acting on the soy substrate. The acid production resulting from the utilization of substrates by LAB strains therefore causes the release of phenolic compounds from food matrices on the soybean starch or polysaccharides. This may be supported by our results that the increased phenolic content was observed when the pH of fermented soymilk was reduced as a result of the acid production after storage for 15 and 20 days (Figure 2C). Our findings were also in agreement with the work of Zhao and shah [17] that concentrations of phenolic acids dramatically increased in soymilk fermented by *L. rhamnosus* and *L. acidophilus* as pH dropped from 6.7 to 4.2. Fernandes et al. [21] evaluated TPC in kefir-fermented soymilk storage and claimed that the storage of soy yogurt caused a decrease in the β-glucosidase content but increased the content of isoflavones. Moreover, Shori and Baba [19] reported that a significant increase in TPCs during storage occurred in all bio-yogurts with or without the addition of the herb cinnamon.

In comparison with other previously published works, Sharma et al. [7] revealed the enhancement of antioxidant activities of the fermented buttermilk and soymilk incubated at 37 °C for 24 h by *P. acidilactici* BD16 (*alaD^+^*). The levels of antioxidant activity were increased from 27.2% to 67.3% and 24.3% to 44.7%, respectively. This was also in agreement with the results showing that the TPC of the buttermilk and soymilk increased upon fermentation using *P. acidilactici* BD16 (*alaD^+^*) by 1.2- and 2-fold, respectively. Marazza et al. [22] reported that soymilk fermentation using *Lactobacillus* spp. enhanced %DPPH inhibition up to 50% when incubated at 37 °C for 48 h compared to that of the unfermented soymilk. Surprisingly, Fakri et al. [23] reported that *Limosilactobacillus fermentum* LAB 10, *Lactiplantibacillus plantarum* LAB 11, and *P. acidilactici* LAB 5 strains could enhance %DPPH inhibition up to 100% while TPC was increased only 10% in the fermented soymilk. Additionally, Song et al. [24] demonstrated that fermented soybean by *Lactiplantibacillus plantarum* contained an increased TPC by 87% in comparison to that of unfermented soybean. Sharma et al. [7] suggested that an increase in the antioxidant potential of various LAB fermented drinks could be attributed to the higher isoflavones content protecting the cells from the detrimental effects of free radicals. Marazza et al. [25] also found that aglycones generated during the fermentation of soymilk by *Lacticaseibacillus rhamnosus* CRL981 acted as hydrogen donors that scavenged DPPH radicals. They also recommended that higher levels of TPC in antioxidative compounds such as phenolics and flavonoids were responsible for enhancing the free radical scavenging capability of fermented drinks. However, Rekha and Vijayalakshmi [26] found that few LAB strains, such as *L. acidophilus*, *L. delbrueckii* subsp. *bulgaricus*, and *L. casei*, have been reported to reduce the TPC of soymilk with an increased fermentation time. Therefore, a discrepancy of increases and decreases in TPC and antioxidant activity of fermented soy products by LAB depends upon probiotic strains.

#### 3.3.2. Viable Cell Counts and Post Acidification

For guaranteed health benefits, the probiotics should be resistant to acidity and should remain viable during the product storage. The viability of probiotic bacteria can be affected by pH, which is a critical environmental factor because it can effectively decrease enzyme activity, and cell growth may be impaired under cold temperatures leading to a notable loss in cell viability [27]. As shown in Figure 2C, the viable cells in soymilk fermented by *P. acidilactici* F3 dropped from 9.7 × 108 to 8.7 × 10^8^ CFUs/mL after 5 days of storage and then remained stable till 20 days in a refrigerated temperature. Besides that, the acidity of the soymilk fermented by *P. acidilactici* F3 gradually decreased from 5.1 to 4.7 after refrigeration for 20 days, as shown in Figure 2C. The decrease in the pH value may be due to the remaining metabolic activity of the F3 strain in the finished product [28]. According to Shori and Baba [19], the reduction in pH probably resulted in the accumulation of acetic acid, citric acid, butyric acid, acetaldehyde, formic acid, and lactic acids by the breakdown of sugar during storage. The results suggest that *P. acidilactici* F3 has a good resistance to cold and was able to survive under acidic pH, thus providing better benefits to consumers when the shelf life of the finished product may be prolonged, making the product quality still acceptable. Low temperatures during fermentation and storage limit cellular activity and metabolism to allow for the minimum energy loss. Consequently, the *P. acidilactici* F3 cell may be more stable under long-term storage conditions. The beneficial effects of probiotics on consumers’ health status were recommended with the minimum probiotic values of 10^6^ to over 10^7^ or 10^8^ CFUs/mL present at the time of consumption [29]. Barbosa et al. [30] found that less than 1 log unit reduction (>10 CFUs/mL) of the viable count of *P. acidilactici* HA-6111-2 (1.8 × 10^8^ CFUs/mL) was recorded after 60 days of storage at 4 °C in concentrated cultures containing reconstituted skim milk by spray drying. The high viable cell counts with a low reduction rate in the fermented soymilk after storage at low temperature suggested that soymilk contains all the vitamin B requirements such as thiamin, riboflavin, biotin, and folic acid for the growth and cell maintenance of *Lactobacillus*, *Bifidobacterium*, and other LAB strains including *Pediococcus* [12]. Ribeiro et al. [31] claimed that *P. acidilactici* B14 demonstrated a survival rate of 45.9% at pH 2.5; 72.4% in 0.3% bile salts; and 95.8% after gastrointestinal transit at pH 4.0. These findings indicate that *P. acidilactici* possesses the ability to survive in harsh conditions during food processing and the prolonged shelf life of finished products.

#### 3.3.3. Organic Acid Production

The concentrations of organic acids such as lactic and acetic acids in soymilk fermentation are shown in Figure 2D. Under the optimum condition, *P. acidilactici* F3 produced principally lactic acid at 1.89 g/L and lower amounts of acetic acid at 0.18 g/L in the ratio 10:1 after 18 h. Similarly, the molar ratio of lactate–acetate was reported at 11.9:1.0 throughout the 24 h fermentation period by *L. rhamnosus* CRL981 [32]. Lactic and acetic acids contributed to lower pH in soymilk fermentation during the storage period (Figure 2D). This suggested that *P. acidilactici* F3 still fermented carbohydrates’ sources during storage to produce energy for survival and to maintain metabolic activities during storage by accumulating both lactic and acetic acids. Additionally, the increase in the acid production confirmed the release of phenolic compounds from food matrices, thus resulting in the significant elevation of antioxidant activity in the fermented soymilk after 15 days of storage.

### 3.4. Antibacterial Activity in Fermented Soymilk during Storage

Probiotic bacteria produce organic acids, hydrogen peroxide, and bacteriocins as antimicrobial substances that suppress the multiplication of pathogens. Lactic and acetic acids account for over 90% of organic acids produced. The lowering of pH due to lactic acid or acetic acid produced by these bacteria in the gut has bactericidal or bacteriostatic effects against many pathogens. *P. acidilactici* has the capacity to produce antimicrobial peptide (pediocin), anti-listerial activity, and an antimicrobial spectrum [5]. Our study showed the antimicrobial activity of *P. acidilactici* F3 against selected pathogens including *E. coli* ATCC 25922, *S.* Typhimurium ATCC 13311, and *S. aureus* ATCC 25923 by co-incubating each of them with fresh or refrigerated fermented soymilk at 37 °C for 24 h. A temperature of 37 °C was used to investigate the survival of pathogens and the probiotic strain in the fresh fermented soymilk after storage at a cold temperature. This reflected the direct application when the product was consumed by consumers while the product passed through out the digestion system at 37 °C.

As shown in Table 3, *P. acidilactici* F3 presenting in fresh and refrigerated fermented soymilk had capability to effectively inhibit growth against *S.* Typhimurium ATCC 13311 followed by *E. coli* ATCC 25922 and *S. aureus* ATCC 25923. No pathogen colony was found on the selective plates for fermented soymilks at D0 to D20 co-incubated with *S.* Typhimurium ATCC 13311 and for fermented soymilks at D10 to D20 co-incubated with *E. coli* ATCC 25922. This confirmed the stability of antimicrobial substances in fresh and refrigerated fermented soymilks. *E. coli* ATCC 25922 was not inhibited by the fresh fermented soymilk (D0) and the storage fermented soymilk (D5) but was completely inhibited by the fermented soymilk storage for 10, 15, and 20 days. This may be due to the fact that the pH values of the fermented soymilks on 0 and 5 days of storage were not as low as the acidic spectrum that may inhibit the growth of *E. coli* since the pH values decreased during the storage period (Figure 2C). The probiotic strain’s ability to inhibit the pathogenic growth in the fermented soymilk against *S.* Typhimurium ATCC 13311 and *E. coli* ATCC 25922 was very impressive but was not effective against *S. aureus* ATCC 25923. However, the probiotic fermented soymilk did not allow for a significant increase in cell concentrations of all three pathogens compared to those of non-fermented soymilk. The results suggested that the fermented soymilk had a positive effect on controlling or even inhibiting the growth of some pathogens and also provided an effective food vehicle to deliver *P. acidilactici* F3 into the GI tract of consumers, thus conferring probiotic effects with long-term application.

### 3.5. Dynamic Inhibition of Pathogens Co-Cultured with Refrigerated Fermented Soymilk

Up to now, limited publications have existed on the antimicrobial activity of *P. acidilactici*. Ribeiro et al. [31] revealed that *P. acidilactici* B14 inhibited antagonism against *E. coli* ATCC 25922, *Bacillus cereus* ATCC 33018, *S. aureus* ATCC 6538P, and *Salmonella* sp. Furthermore, Abbasiliasi et al. [33] introduced *P. acidilactici* Kp10, which exhibited antimicrobial activity against several Gram-positive and Gram-negative food-spoilage and food-borne pathogens, including *Listeria monocytgenes* ATCC 15313, *Salmonella enterica* ATCC 13311, *Shigella sonnei* ATCC 9290, *Klebsiella oxytoca* ATCC 13182, *Enterobacter cloacae* ATCC 35030, and *Streptococcus pyogenes* ATCC 12378. Additionally, *Enterococcus faecium* NCIM 5423 and *L. plantarum* Acr2, except for *P. acidilactici* NCIM 5424, could inhibit the growth of *Listeria monocytogenes* ScottA after co-cultivation for 6 h [6]. The monograph from SEM analysis revealed that bacteriocin produced by LAB strains damaged cell membranes of pathogens as indicated by the shrinkage or disruption of cells. However, they reported antimicrobial activity against all pathogens using the supernatant of the MRS culture broth tested by the agar diffusion method. The information of whether the application of *P. acidilactici* in finished products as a vehicle delivering the microbe could be applied against food-borne pathogens was missing. Unlike previous works, this work demonstrated the good growth inhibition of pathogens in the dynamic growth system during co-cultivation of *P. acidilactici* F3 and an individual pathogen. *P. acidilactici* F3 possessed high antimicrobial activity against Gram-negative bacteria such as *S.* Typhimurium ATCC 13311 and *E. coli* ATCC 25922 but less against Gram-positive bacteria like *S. aureus* ATCC 25923.

**Figure 3:**
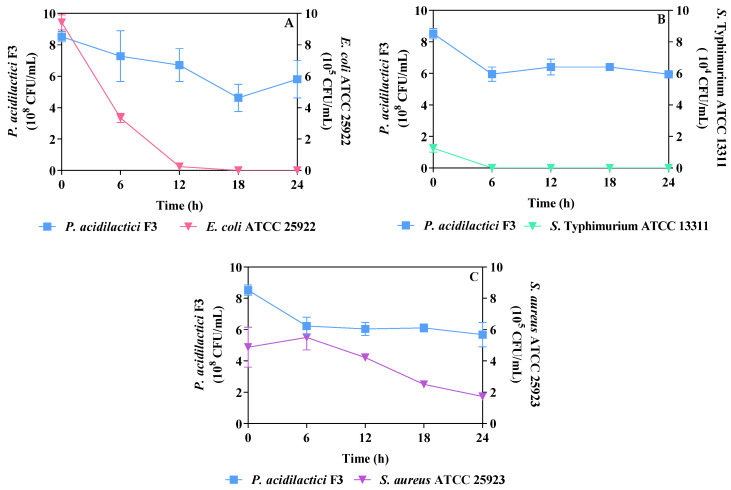
Antimicrobial activity against (**A**) *E. coli* ATCC 25922, (**B**) *S.* Typhimurium ATCC 13311, and (**C**) *S. aureus* ATCC 25923 co-incubated at 37 °C for 24 h with 20-day-refrigerated soymilks fermented by *P. acidilactici* F3.

As shown in Figure 3, the number of *P. acidilactici* F3 cells dropped slightly when co-incubated with each pathogen. Although, viable *P. acidilactici* F3 cells from 20-day-refrigerated fermented soymilk presented at levels of 5.68–6.00 × 10^8^ CFUs/mL throughout the 24 h co-incubation with each pathogen at 37 °C. Additionally, *P. acidilactici* F3 in fermented soymilk completely inhibited the growth of *E. coli* ATCC 25922 after 18 h (Figure 3A) and *S.* Typhimurium ATCC 13311 after a 6 h (Figure 3B) co-incubation time at 37 °C. This may result from the dominant occupation or synergistic effect of viable cells of *P. acidilactici* F3, organic acids, acidic pH, and antimicrobial compounds, thus providing adverse effects on the growth of both pathogens.

Results in this study also confirmed that the produced lactic and acetic acids in the fermented soymilk (pH 4.7) and the additional acid production by *P. acidilactici* F3 during co-cultivation could completely inhibit the growth of *E. coli* strains. Most *E. coli* strains were sensitive to lactic acid and acetic acid. The minimum pH of acetate above 5.5 allowed *E. coli* to grow in adjusted tryptic soy broth. Oh et al. [34] stated the effect of organic acids on the survival of *E. coli* under the minimum inhibitory pH values of acetic acid and lactic acid at 5.0 and 4.0, respectively. Unfortunately, the soymilk fermented by *P. acidilactici* F3 had less antagonism against the *S. aureus* pathogen. However, viable cells of *S. aureus* gradually decreased versus the fermentation time (Figure 3C). Changes in pH of the fermented soymilk during storage were in the range of 5.1 to 4.7 (Figure 2C), therefore, this may allow for the survival of *S. aureus* under these pH values. Abd EI-Gawad et al. [28] also revealed that the growth of *S. aureus* occurred at pH 4.6 but not at pH 4.3, in which *S. aureus* was still detected in probiotic soymilk fermented by *Bifidobacterium* Bb-12 and Bb-46.

Besides the candidate pathogens used in this study, *Bacillus cereus* is the known source of food-borne illnesses associated with an enterotoxin contaminating foods prior to eating or even those produced by the pathogen inside the host after ingestion. *B. cereus* and its toxin can easily spread to many types of fresh and processed foods, including meat, cereal, vegetables, milk, and soups. Additionally, *Listeria monocytogenes*, a species of pathogenic bacteria, causes a disease called listeriosis when people eat food or drink water contaminated with the bacteria. This species can be widely spread in environments during the transportation of harvested, packed, and processed food. Thus, applications of probiotics to prevent host infections of these two species are currently attractive. Many studies recently showed that the cultured broth of *P. acidilactici* could inhibit the growth of both species. For example, *P. acidilactici* P12 culture and its cell-free filtrate showed a significant potential inhibition of biofilm formation of *B. cereus* M50 on different food-manufacturing surfaces [35]. The MRS-cultured broth of *P. acidilactici* Gbf48 and Gbf50 contained the antimicrobial effect against *B. cereus* and *L. monocytogenes* [36]. Diguta et al. [37] revealed that the fresh suspension of *Pediococcus* spp. isolated from Kombucha consortium had high inhibitory activity against *B. cereus* and *L. monocytogenes.* Kaya and Simsek [38] also showed that *P. acidilactici* PFC69 decreased viable cells of *B. cereus* ATCC 11778 in tarhana dough. Rwubuzizi et al. [39] isolated *P. pentosaceus* STO5BG producing bacteriocin against *Listeria* spp. It is therefore interesting to study further the antimicrobial activities of soymilk fermented by *P. acidilactici* F3 against *B. cereus* and *L. monocytogenes*. This information will bring more insight for applying *P. acidilactici* F3 as a potential probiotic strain against a broad-range spectrum of food-borne pathogens in the form of free cells or cells associated with food matrices, not limited to fermented soymilk.

It is generally known that the production of bioactive molecules, including peptides, and phenolic compounds is enhanced in soymilk fermented by LAB. The bioactive molecules provide several health benefits including antihypertensive, antitumor, antidiabetic, and antihyperlipidemic effects [40]. This brings attention to the development of fermented functional soy products, dietary supplements, and even pharmaceuticals intending to provide specific health benefits. In this study, optimized parameters offering the highest antioxidant activity in the soymilk fermented by *P. acidilactici* F3 were elucidated. With these optimized conditions, the fermented soymilk still possessed high viability of the F3 strain up to 8 log CFUs/mL during storage for 20 days under high acidity. This was unlike other reports using *Lactobacillus* species to ferment soymilk, in which its viability dropped during storage [12]. Additionally, our results suggest that the stability of the scavenging activity, TPC level, and antimicrobial activity against food-borne pathogens in the soymilk fermented by *P. acidilactici* F3 ensures the same product quality along the whole 3-week shelf life, guarantees the retention of nutritional values, and benefits from both antioxidant and antimicrobial activities to give consumers high confidence.

## 4. Conclusions

*P. acidilactici* F3 was potentially capable of producing a high antioxidant activity by fermenting soymilk. The development of the fermentation *P. acidilactici* F3 culture was optimized by supplementing 5 g/L glucose in soymilk, with incubation at 30 °C with a shortened fermentation time from 24 h to 18 h. With this condition, higher antioxidant properties up to value of 78.9% DPPH inhibition were attained, which was up to a 23% increase comparable to that of unfermented soymilk. Product stability was evaluated for changes in physical and biological properties of fermented soymilk during storage. Antioxidant activity and viable cells were stable when there were reductions in pH paralleled with increases in organic acids, total phenolic contents, and antimicrobial activity after 20 days of storage. Particularly, the fermented soymilks inhibited pathogens including *S.* Typhimurium ATCC 13311 and *E. coli* ATCC 25922 after co-culture incubation at 37 °C. Hence, soymilk fermented by *P. acidilactici* F3 may be a good source of the probiotic strain that potentially enhances antioxidant capacity and antimicrobial activity with high product stability along a prolonged shelf life for three weeks. With these properties of the soymilk fermented by *P. acidilactici* F3, it confirms the retention of nutritional values to consumers with high confidence. Additionally, the results will bring further insight for applying *P. acidilactici* F3 as one of the potential probiotic sources in other food matrices, not limited to fermented soymilk.

## Figures and Tables

**Table 1:** Parameters and their levels for optimizing antioxidant activity production using RSM methodology during soymilk fermentation by the *P. acidilactici* F3 strain.

Parameters	Level				
	−1.4142	−1	0	1	1.4142
Cell inoculum (%, *v*/*v*)	0.58	1	2	3	3.41
Temperature (°C)	15.85	20	30	40	44.14
Fermentation time (h)	4.68	8	16	24	27.31
Glucose (g/L)	1.46	2.5	5	7.5	8.53

**Table 2:** Experimental design of RSM strategy using CCD methodology for optimizing %DPPH.

Run	Parameters				%DPPH Activity	
	A: Inoculum (%, *v*/*v*)	B: Temperature (°C)	C: Time (h)	D: Glucose (g/L)	Observed (%)	Predicted (%)
1	2	15.85	16	5	71.6 ± 1.6	71.6
2	2	30	16	1.46	77.7 ± 0.8	77.2
3	1	40	8	7.5	72.8 ± 0.9	70.6
4	2	30	27.31	5	81.1 ± 0.2	78.9
5	0.58	30	16	5	74.9 ± 0.4	73.3
6	2	44.14	16	5	72.7 ± 0.8	74.6
7	3	40	24	2.5	72.7 ± 0.1	74.4
8	3	40	8	2.5	76.8 ± 0.3	76.6
9	3.41	30	16	5	72.9 ± 0.3	72.9
10	1	40	24	7.5	75.9 ± 1.2	76.3
11	3	20	24	7.5	66.9 ± 1.2	70.6
12	1	20	8	2.5	61.7 ± 0.5	63.4
13	3	20	8	7.5	68.6 ± 0.8	68.4
14	2	30	16	7.5	77.1 ± 0.9	77.2
15	2	30	4.68	5	76.3 ± 1.6	76.8
16	2	30	16	8.53	72.0 ± 1.1	74.9
17	2	30	16	7.5	77.1 ± 0.9	77.2
18	1	20	24	2.5	63.9 ± 0.1	63.7

**Table 3:** Antimicrobial activity against pathogens by *P. acidilactici* F3 fermented soymilk.

Treatment	Viability Count after 24 h Co-Incubation Time at 37 °C (CFUs/mL)					
	Soymilk	D0	D5	D10	D15	D20
**Anti-*E. coli*** *P. acidilactici* F3 *E. coli* ATCC 25922	ND 2.80 × 10^8^	7.26 × 10^8^ 1.01 × 10^5^	5.25 × 10^8^ 3.65 × 10^4^	4.73 × 10^8^ ND	5.10 × 10^8^ ND	5.81 × 10^8^ ND
**Anti-*S.* Typhimurium** *P. acidilactici* F3 *S.* Typhimurium ATCC 13311	ND 4.04 × 10^8^	7.50 × 10^8^ ND	4.12 × 10^8^ ND	6.10 × 10^8^ ND	5.10 × 10^8^ ND	6.00 × 10^8^ ND
**Anti-*S. aureus*** *P. acidilactici* F3 *S. aureus* ATCC 25923	ND 1.97 × 10^8^	3.25 × 10^8^ 4.40 × 10^5^	8.87 × 10^8^ 7.07 × 10^5^	6.56 × 10^8^ 1.97 × 10^5^	5.10 × 10^8^ 4.40 × 10^5^	5.68 × 10^8^ 1.73 × 10^5^

ND = not detected; D = days after storage of the fermented soymilk at 4 °C.

## Data Availability

The original contributions presented in the study are included in the article, and further inquiries can be directed to the corresponding authors.
